# TRaCE+: Ensemble inference of gene regulatory networks from transcriptional expression profiles of gene knock-out experiments

**DOI:** 10.1186/s12859-016-1137-z

**Published:** 2016-06-24

**Authors:** S.M. Minhaz Ud-Dean, Sandra Heise, Steffen Klamt, Rudiyanto Gunawan

**Affiliations:** Institute for Chemical and Bioengineering, ETH Zurich, Zurich, Switzerland; Swiss Institute of Bioinformatics, Lausanne, Switzerland; Max Planck Institute for Dynamics of Complex Technical Systems, Magdeburg, Germany

**Keywords:** Gene regulatory network, Network inference, Design of experiments, Signed directed graph, Transitive reduction

## Abstract

**Background:**

The inference of gene regulatory networks (GRNs) from transcriptional expression profiles is challenging, predominantly due to its underdetermined nature. One important consequence of underdetermination is the existence of many possible solutions to this inference. Our previously proposed ensemble inference algorithm TRaCE addressed this issue by inferring an ensemble of network directed graphs (digraphs) using differential gene expressions from gene knock-out (KO) experiments. However, TRaCE could not deal with the mode of the transcriptional regulations (activation or repression), an important feature of GRNs.

**Results:**

In this work, we developed a new algorithm called TRaCE+ for the inference of an ensemble of signed GRN digraphs from transcriptional expression data of gene KO experiments. The sign of the edges indicates whether the regulation is an activation (positive) or a repression (negative). TRaCE+ generates the upper and lower bounds of the ensemble, which define uncertain regulatory interactions that could not be verified by the data. As demonstrated in the case studies using *Escherichia coli* GRN and 100-gene gold-standard GRNs from DREAM 4 network inference challenge, by accounting for regulatory signs, TRaCE+ could extract more information from the KO data than TRaCE, leading to fewer uncertain edges. Importantly, iterating TRaCE+ with an optimal design of gene KOs could resolve the underdetermined issue of GRN inference in much fewer KO experiments than using TRaCE.

**Conclusions:**

TRaCE+ expands the applications of ensemble GRN inference strategy by accounting for the mode of the gene regulatory interactions. In comparison to TRaCE, TRaCE+ enables a better utilization of gene KO data, thereby reducing the cost of tackling underdetermined GRN inference. TRaCE+ subroutines for MATLAB are freely available at the following website: http://www.cabsel.ethz.ch/tools/trace.html.

## Background

The central dogma of molecular biology describes the process by which genetic information flows linearly from deoxyribonucleic acid (DNA) to ribonucleic acid (RNA) to proteins through the process of transcription and translation [1]. This dogma has guided research on the causes of cellular phenotype and diseases since its inception in 1956. However, such reductionist view has been continually challenged in the post-genomic era, during which we also saw the rise of systems biology and the use of networks to understand biology at all levels. One prominent example of such networks is a gene regulatory network (GRN), which describes how the protein product(s) of one gene activates or inhibits the transcription of other genes. The knowledge of this network and its pathological alterations could lead to, among other things, a better understanding of how cell phenotype arise from the interactions among cell constituents, and to novel treatments and drugs for genetic diseases such as cancer [2].

The inference of GRN has received great attention from the systems biology community, especially with the ubiquity of gene transcriptional profiling using DNA microarray chip and RNA sequencing. A multitude of network inference methods now exist in the literature for the identification of GRN structure from gene transcriptional expression data [3–9]. These methods adapted concepts and techniques from multiple disciplines such as information theory, statistics, machine learning and systems theory. The large number of methods called for the establishment of gold standard data for a fair and objective assessment. The DREAM (Dialogue for Reverse Engineering, Assessment and Methods) project materialized as an answer [10], and the GRN inference became a topic in several community-wide challenges within this project.

The results of several GRN inference challenges signified a fundamental issue in this inference. For example, many of the algorithms employed by participants in DREAM 4 *in silico* network inference challenge did not perform well in inferring small to moderately-sized GRNs (10–100 genes). This poor performance was observed despite the availability of transcriptional expression data of all genes from the complete set of single-gene knock-out (KO) experiments, i.e. deleting each and every gene, one at a time [8, 9, 11]. The network inference challenge further showed that distinguishing direct and indirect regulations was one of the common weaknesses among the participating methods. As we demonstrated recently [12], these difficulties arose because the above GRN inference problems were underdetermined, i.e. the GRNs were not inferable and there could in fact exist an ensemble of GRN structures that agreed with the gene KO data.

We previously developed TRaCE (Transitive Reduction and Closure Ensemble) for constructing an ensemble of GRN structures that are consistent with the input transcriptional expression data of all genes in the network from gene KO experiments [12]. This ensemble represented the uncertainty in the GRN inference. In developing TRaCE, we employed directed graphs to model the GRNs, where nodes in such graphs describe the genes and directed edges describes the gene regulations. Following the GRN inference formulation in DREAM challenges, we previously ignored the signs of the edges and sought only to establish the existence of gene regulations. Nevertheless, the edge signs in a GRN digraph are often of great interest and significance as they indicate the modes of the gene regulations. Here, a positive edge reflects an activation, while a negative edge describes a repression. Several notable network inference algorithms such as TRANSitive reduction for WEighted Signed Digraphs (TRANSWESD) [13] and Local Transitive Reduction (LTR) [14] previously considered the inference of GRN digraph with signed (and weighted) edges. However, these algorithms were not designed for inferring an ensemble of GRN structures.

In this work, we addressed the aforementioned drawback of TRaCE by developing a new ensemble inference method, called TRaCE+. The new method uses a signed digraph model of the GRN, i.e. the edges have signs. Like TRaCE, TRaCE+ generates the upper and lower bounds of the ensemble, but in the form of signed digraphs. The ensemble bounds from TRaCE+ are also compatible with our recent optimal design of gene KO strategy called REDUCE [15]. We demonstrated the advantages of TRaCE+ over TRaCE in the ensemble inference of *Escherichia coli* GRN and in the iterative inference of 100-gene gold standard GRNs from DREAM 4 *in silico* network inference challenge.

## Methods

### Definitions

In this section we provide basic concepts of graph theory that are relevant to the development of TRaCE+. A *graph G* is defined by the pair (*V*(*G*)*,E*(*G*)) where *V*(*G*) denotes the set of *vertices* or *nodes* and *E*(*G*) ⊆ *V*(*G*) × *V*(*G*) denotes the set of *edges*. The number of nodes *n*(*G*) and edges *m*(*G*) are called the *order* and *size* of the graph, respectively. In a *directed graph*, an edge is defined by an ordered pair of nodes (*i,j*) denoting the edge direction, pointing from node *i* to node *j*. Here, node *i* is called a *parent* of node *j*, while node *j* is called a *child* of node *i*. The edge (*i,j*) is also said to be incident to nodes *i* and *j.* Finally, a *signed digraph G*^+^ = (*V*(*G*^+^), *E*(*G*^+^), *S*(*G*^+^)) is the digraph (*V*(*G*^+^), *E*(*G*^+^)) with an edge mapping *S* : *E* → {+,−} that assigns a positive or negative sign to each edge.

A *directed path* in a digraph *G* is a sequence of nodes *v*_1_, *v*_2_, *v*_3_, … *v*_*n*_ such that (*v*_1_, *v*_2_), (*v*_2_, *v*_3_), … (*v*_*n* − 1_, *v*_*n*_) ∈ *E*(*G*). The number of edges in a directed path is called the *path length*. The first node of a directed path is called the *start node*, and the last node is called the *end node*. A *directed cycle* is a directed path where the start node and the end node are the same. A *directed acyclic graph* (DAG) is a digraph which does not contain any directed cycle. A node *v* is said to be *accessible* from another node *u* if there exist a directed path from node *u* to node *v*. In this case, node *u* is an *ancestor* of node *v*, and node *v* is a *descendant* of node *u*. The *adjacency matrix* of a digraph *G* is a *n*(*G*) × *n*(*G*) matrix where the (*u,v*)-th element is 1 if there exists a directed edge from node *u* to node *v* in *G*, and 0 otherwise. Meanwhile, the *accessibility matrix* of a digraph *G* is a *n*(*G*) × *n*(*G*) matrix where the (*u,v*)-th element is 1 if there exists a directed path from node *u* to node *v* in *G*, and 0 otherwise. Multiple digraphs can have the same accessibility matrix, among which the digraph with the fewest edges is called the *transitive reduction.*

**Fig. 1:**
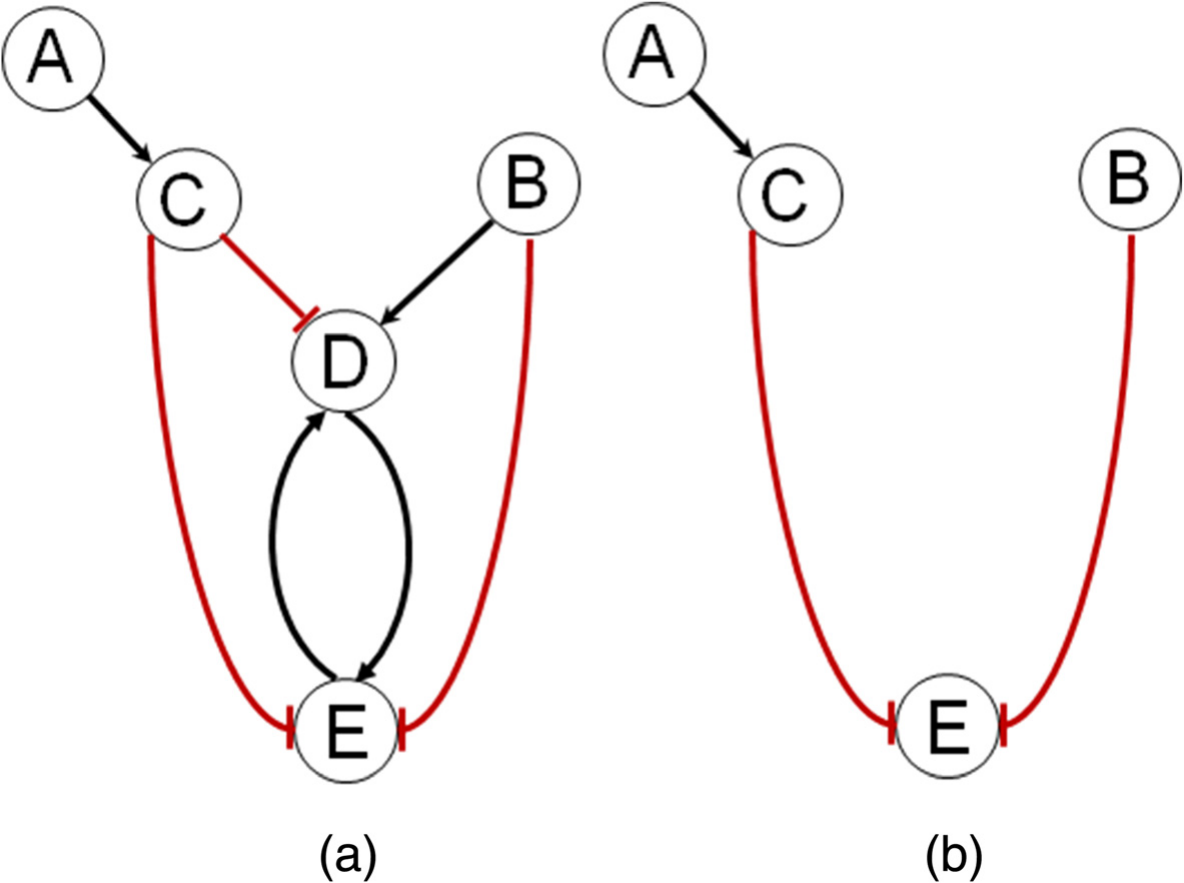
Illustration of signed digraph GRNs. **a** Example of a signed digraph ${G}_{\varnothing}^{+}$. The pointed black arrows indicate positive regulations while the flathead red arrows represent negative regulations. **b** The network ${G}_{\left\{\mathrm{D}\right\}}^{+}$ in which gene D has been deleted

In the following sections, we focus on the inference of GRN structure in the form of a signed digraph. We denote the GRN of interest as ${G}_{\varnothing}^{+}$, which is also referred to as the wild-type GRN. In such a graph, the nodes represent the genes, and the signed directed edges indicate the gene regulations. A positive (negative) edge pointing from gene *i* to gene *j* implies that the products of gene *i* upregulates (represses) the expression of gene *j*. Figure 1a gives an example of a signed digraph of a GRN with 5 genes (*n*(*G*) = 5) and 7 gene regulatory edges (*m*(*G*) = 7). Three of the gene regulations are negative (repressions), while four are positive (upregulations). Here, gene *C* is a parent of genes *D* and *E,* while gene *D* is a child of genes *C, B* and *E*. Meanwhile, gene *A* is an ancestor of the genes *C, D* and *E*, while gene *E* is a descendant of the genes *A, B, C* and *D*. We further denote the GRN corresponding to knocking-out (deleting) a set of genes ${V}_{KO}\subset V\left({G}_{\varnothing}^{+}\right)$ by ${G}_{V_{KO}}^{+}$. Figure 1b illustrates the network ${G}_{\mathrm{D}}^{+}$, where all edges incident to gene *D* have been deleted from the GRN ${G}_{\varnothing}^{+}$ in Fig. 1a.

### TRaCE+

An important consequence of the underdetermined nature of GRN inference is the existence of multiple solutions to the inference problem. In order to deal with this consequence, we previously developed an ensemble inference algorithm called TRaCE [12]. In the development of TRaCE and TRaCE+, we recognized a fundamental limitation in using steady state transcriptional expression data of gene KO experiments for GRN inference. This limitation relates to the inability to differentiate direct and indirect gene regulations from such expression data. For example, we expect that knocking out a transcription factor (TF) gene *u* would lead to steady-state differential expressions among genes that directly regulated by that TF, as well as genes that are indirectly regulated by *u* through the involvement of other TFs. Following the definitions above, genes which show differential expression upon knocking out gene *u* thus correspond to all nodes that are accessible from node *u*. Given steady state transcriptional expression data from the complete set of single-gene KOs, one could obtain at most the accessibility relationship (matrix) among the genes.

In TRaCE, we seek to identify the ensemble (family) of GRN digraphs that are consistent with steady state differential gene expressions in KO experiments. The true GRN is thus expected to belong to this ensemble. More specifically, TRaCE generates two digraphs: the ensemble upper bound *G*^*U*^ and the ensemble lower bound *G*^*L*^, which compactly represent the entire ensemble. The upper bound *G*^*U*^ represents the largest digraph in the ensemble, i.e. one with the most gene regulations or edges. Edges in *G*^*U*^ may include both direct and indirect gene regulations. Meanwhile, the lower bound *G*^*L*^ is constructed from *G*^*U*^ by removing all edges which can be explained by an indirect gene regulation. Therefore, any GRN in the ensemble must contain all edges in the lower bound *G*^*L*^ and may include some or all the edges in the upper bound *G*^*U*^*.* The set of gene regulatory edges that belong to the upper bound *G*^*U*^ but are missing from the lower bound *G*^*L*^, denoted by *E*_*U*_, are appropriately called uncertain edges. These edges are uncertain since their existence could not be verified by the available data. Furthermore, the number of uncertain edges reflects the degree of uncertainty in the GRN inference. Importantly, by knowing which edges are uncertain, we could optimize KO experiments in order to maximize the number of edge verifications [15].

One of the biggest drawbacks of TRaCE is its disregard of the signs (modes) of the regulatory edges. For this reason, we have developed TRaCE+. Like TRaCE, TRaCE+ generates the upper and lower bounds of the ensemble, but with signed edges (i.e., these bounds are signed digraphs). TRaCE+ comprises two main algorithms for (1) constructing the ensemble upper and lower bounds using data from the complete set of single-gene KOs, and (2) updating the ensemble bounds using additional gene KO data. Below, we provide more details of the two algorithms.

#### Construction of upper bound from single-gene KOs

In constructing the upper and lower bounds of the ensemble for TRaCE+, we start by putting together the accessibility matrix using steady state transcriptional expression data from the complete set of single-gene KOs. As mentioned above, the accessibility matrix includes both direct and indirect gene regulations, and thus provides an upper bound for the GRNs in the ensemble. Here, we follow the same procedure as that in TRaCE [12]. Briefly, for each technical replicate, we obtain the sample mean ${\mu}_j^{\hbox{'}}$ and standard deviation ${s}_j^{\hbox{'}}$ of the expression of gene *j*. Subsequently, we calculate the corrected sample mean *μ*_*j*_ and standard deviation *s*_*j*_ by excluding the expression data of gene *j, g*_*ij*_ that differ from ${\mu}_j^{\hbox{'}}$ by more than ${z}_{cutoff}{s}_j^{\hbox{'}}$. Using *μ*_*j*_ and *s*_*j*_, we then compute the z-scores $z\left(i,j\right)=\frac{g_{i,j}-{\mu}_j}{s_j}$ which indicates the differential expression of gene *j* in the KO of gene *i.* Finally, we average the z-scores over the technical replicates to give the overall z-score matrix *Z*(*i,j*), based on which we obtain the accessibility matrix by the following criteria:

$$ Acc\left(i,j\right)=\left\{\begin{array}{c}\hfill 1\hfill \\ {}\hfill 0\hfill \end{array}\begin{array}{c}\hfill \kern1.5em \mathrm{if}\hfill \\ {}\hfill \kern1em \mathrm{if}\hfill \end{array}\right.\kern1em \begin{array}{c}\hfill \left|Z\left(i,j\right)\right|>{Z}_{threshold}\hfill \\ {}\hfill \left|Z\left(i,j\right)\right|\le {Z}_{threshold}\hfill \end{array} $$

In this study, we use the accessibility matrix from the single-gene KO data as the initial ensemble upper bound *G*^*U*^, which will later be refined by incorporating steady state gene expression data from optimally designed KO experiments. In the case studies, we employed *z*_*cutoff*_ = 3 and *z*_*threshold*_ *=* 2 following the recommendations in TRaCE [12]. In contrast to TRaCE, we also set a sign for each edge (non-zero element in *Acc*) in the upper bound *G*^*U*^ as follows:

$$ S\left(i,j\right)=\left\{\begin{array}{c}\hfill +\hfill \\ {}\hfill -\hfill \end{array}\kern1.5em \begin{array}{c}\hfill \mathrm{if}\hfill \\ {}\hfill \mathrm{if}\hfill \end{array}\kern1.5em \begin{array}{c}\hfill Z\left(i,j\right)\le 0\hfill \\ {}\hfill Z\left(i,j\right)>0\hfill \end{array}\right. $$

#### Construction of initial lower bound

In TRaCE, the lower bound of the ensemble *G*^*L*^ came from applying ConTREx (condensation, transitive reduction, and expansion) to the upper bound *G*^*U*^ above without considering the signs [12]. Briefly, the upper bound *G*^*U*^ was first condensed by lumping nodes in directed cycles, to give a DAG of strongly connected components. The subsequent transitive reduction involved removing from this DAG, any edge (*i,j*) for which there exist a directed path from node *i* to node *j* not involving this edge. Finally, in order to get the lower bound *G*^*L*^, we expanded the strongly connected components of directed cycles, during which any edges incident to cycles were removed, except those between nodes in a two-node cycle. Consequently, except when *G*^*U*^ is a DAG, *G*^*L*^ may no longer share the same accessibility matrix as *G*^*U*^. However, the set of uncertain edges defined by the resulting *G*^*U*^ and *G*^*L*^ appropriately include edges in cycles with more than two nodes, as there exist ambiguity in either the existence or the direction of these edges.

Extending the transitive reduction procedure to a signed digraph means that we should remove any edge (*i,j*) for which there exist a directed path from node *i* to node *j* not involving the edge (*i,j*) *and* the cumulative product of the edge signs on this path is equal to the sign of the edge (*i,j*). This simple procedure may not work when *G*^*U*^ contains a *negative cycle* (a directed cycle with an odd number of negative edges), since the cumulative sign of such a cycle alternates depending on how many times one traverses through it. A recent study comparing different ways to obtain the transitive reduction of a signed GRN digraph (with and without cycles) recommended a simple procedure called Local Transitive Reduction (LTR) [14]. In the following, we have adapted LTR to generate the lower bound signed digraph *G*^*L*^ for TRaCE+.

In LTR, an edge (*i,j*) representing the regulation of gene *j* by gene *i*, is removed when the effect of gene *i* on gene *j* can be explained by an indirect regulation involving at least one other gene. Such indirect regulation should explain not only the mode of the gene regulation (i.e. the sign of (*i,j*)) but also the strength of the regulation. Here, the strength of gene regulations is quantified by the weighting factor *W*(*i,j*) (for each edge (*i,j*) in *G*^*U*^), which equals to the magnitude of the correlation coefficient between gene *j* and gene *i*, averaged over the technical replicates. In the calculation of *W*(*i,j*), we exclude data from gene *j* KO experiment since the differential expression of gene *j* in this experiment may not reflect the effect of gene *i* on gene *j*.

Adapting the LTR procedure, we first equate the two bounds (*G*^*L*^ *= G*^*U*^), and subsequently remove any edge (*i,j*) in *G*^*L*^ for which there exists a path of length 2 in *G*^*U*^ explaining the edge (*i,j*), or more precisely there exists a node *k* with (*i*, *j*), (*i*, *k*), (*k*, *j*) ∈ *G*^*U*^ and *k* ∉ {*i*, *j*}, such that

- the directed path *i,k,j* is sign consistent with (*i,j*), i.e. *S*(*i*, *j*) = *S*(*i*, *k*)*S*(*k*, *j*).
- the weight of the edge (*i,j*) satisfies *w*_*cut*_*W*(*i*, *j*) < *W*(*i*, *k*)*W*(*k*, *j*), where *w*_*cut*_ ∈ [0, 1] is a cutoff ratio.

The first condition requires the overall sign of the indirect regulation to equal to the sign of the gene regulation (*i,j*). Meanwhile, the second condition requires the overall strength of the indirect regulation, which is assumed to accrue multiplicatively, to exceed a prescribed cutoff fraction of the strength of the edge (*i,j*). Note that by setting *w*_*cut*_ = 0, we effectively ignore the contribution of the edge weights. By considering only directed paths of length 2, the procedure above avoids the problem associated with negative cycles since any path going through a cycle more than once would necessarily have a length longer than 2. In addition, LTR does not require condensation and expansion steps as in TRaCE’s ConTREx. Unlike the original version of LTR, here we do not check whether a removal of an edge would change the outcome of previous edge removals, and as a result, *G*^*L*^ may not have the same accessibility relationships as *G*^*U*^*.* While implementing the check would lead to fewer uncertain edges, it would also cause more false positive errors that could neither be corrected by additional data nor new experiments (see Discussion).

**Fig. 2:**
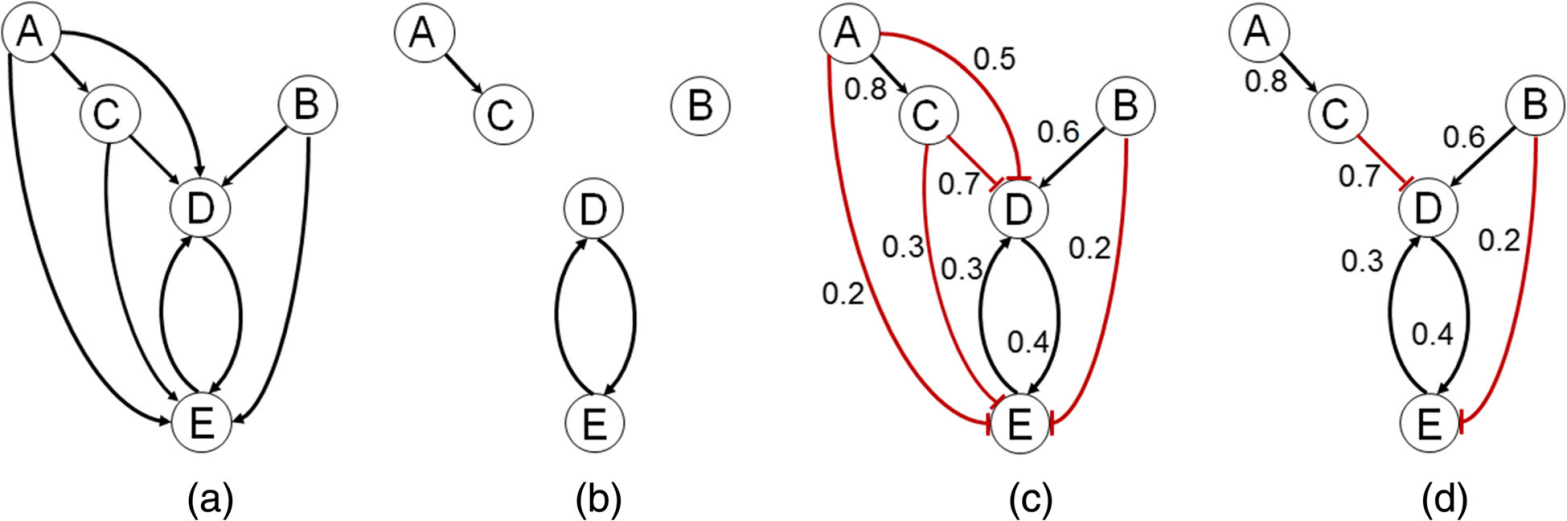
Comparison of ensemble upper and lower bounds obtained from single-gene KO data by (**a** and **b**) TRaCE and (**c** and **d**) TRaCE+ (with randomly assigned weights). The true GRN is shown in Fig. 1 **a**. (**a** and **c) ** Ensemble upper bound *G* ^*U*^. (**b** and **d**) Ensemble lower bound *G* ^*L*^. Note that the lower bound from TRaCE is not the transitive reduction of the upper bound due to the existence of a cycle between D and E

Figure 2 illustrates the computations of the lower bound *G*^*L*^ in TRaCE and in TRaCE+ (using *w*_*cut*_ of 0.3) for the wild-type GRN ${G}_{\varnothing}^{+}$ in Fig. 1a. The comparison showed that accounting for signs and weights of the edges could lead to a higher retention of true edges in *G*^*L*^, and thus to fewer uncertain edges. This difference demonstrated that some information could be lost by disregarding edge signs in the GRN inference, as done in TRaCE.

#### Ensemble bounds update

The ensemble bounds update algorithm allows the incorporation of transcriptional expression data from gene KO experiments beyond single-gene KOs. The update follows an iterative procedure involving (1) evaluation of *separatoids* for uncertain edges, (2) determination of verifiable uncertain edges, and (3) refinement of the ensemble bounds. We previously introduced the concept of separatoid based on a simple premise, as illustrated below. Consider the ensemble upper and lower bounds in Fig. 2c and d, which define three uncertain edges (A, D), (A, E) and (C, E). Here, the verification of the uncertain edge (A, D) will be simple if we delete gene C from the network. More specifically, in the background of gene C KO, we can verify the existence and sign of the edge (A, D) based on whether a perturbation to gene A causes a differential expression of gene D in the appropriate direction. In this case, gene C is a separatoid of the uncertain edge (A, D).

More generally, we define a separatoid of an uncertain edge (*i*, *j*) ∈ *E*_*U*_, denoted by *Sep*(*i*, *j*), as the set of nodes whose removal would eliminate any directed path of length 2 or longer from node *i* to node *j* [15]. The deletion of genes in *Sep*(*i*, *j*) would therefore give the GRN *G*_*Sep*(*i*,*j*)_ where the only remaining directed path from gene *i* to gene *j* is the edge (*i*, *j*) itself. Consequently, we can verify the existence of the uncertain edge (*i*, *j*) by assessing whether gene *j* is differentially expressed upon perturbing gene *i* in the background of *Sep*(*i*, *j*) gene deletions. Returning to the ensemble bound in Fig. 2c and d, we can find the separatoids of the uncertain edge (A, E), which are gene C, gene D and the combination of genes C and D; as well as the separatoid of the uncertain edge (C, E), which is gene D.

As illustrated above, an uncertain edge may have more than one separatoid, while several uncertain edges may share a separatoid. Following our previous work [15], in the first step, we compute three separatoids for each uncertain edge (*i*, *j*) ∈ *E*_*U*_:

1. *Sep*_1_(*i, j*) = children of *i* in *G*^*U*^ ∩ ancestors of *j* in *G*^*U,*0^
2. *Sep*_2_(*i, j*) = descendants of *i* in *G*^*U,*0^ ∩ parents of *j* in *G*^*U*^
3. *Sep*_3_(*i, j*) = descendants of *i* in *G*^*U,*0^ ∩ ancestors of *j* in *G*^*U,*0^

where *G*^*U*^ is the most updated upper bound and *G*^*U*,0^ is the initial upper bound from single-gene KO data. The separatoids above are not the only separatoids for the uncertain edge (*i*, *j*). We limit our analysis only to these separatoids because they are easy to compute. Finding all separatoids for a given edge constitutes finding the longest path between two nodes, which is a NP-hard problem [16]. In the illustration above, the three separatoids for the uncertain edge (A, E) are given by *Sep*_*1*_(*A,E*) *=* {*C*}, *Sep*_*2*_(*A,E*) *=* {*D*}, and *Sep*_*3*_(*A,E*) *=* {*C,D*}.

In the second step we identify among the set of input gene KOs **V**_**KO**_ (including single-gene KOs), pairs of KO experiments whose data would allow the verification of the edge (*i,j*). More precisely, for each (*i,j*) ∈*E*_*U*_, we search for the pair of KO experiments $\left({V}_{K{O}_k},{V}_{K{O}_k}\cup i\right)\in {\mathbf{V}}_{\mathbf{KO}}\times {\mathbf{V}}_{\mathbf{KO}}$ such that $Se{p}_l\left(i,j\right)\subset {V}_{K{O}_k}$ for any *l* = 1, 2, 3, and $i,j\notin {V}_{K{O}_k}$. Following the definition of a separatoid above, if gene *j* is differentially expressed between any of such pairs of KO experiments, then we have evidence supporting for the existence of the uncertain edge (*i,j*).

In the third step, for each uncertain edge (*i,j*), we perform a (two-tailed) two-sample *t*-test with *α* = 0.01 to determine whether the expression of gene *j* is significantly different between the KO of ${V}_{K{O}_k}\cup i$ in comparison to the KO of ${V}_{K{O}_k}$. In the case that we only find one pair of such experiments for an uncertain edge (*i,j*), we remove this edge from the upper bound *G*^*U*^ upon a failure to reject the null hypothesis in the *t*-test above. Otherwise, we add the edge (*i,j*) to the lower bound *G*^*L*^. Further, if the average expression of gene *j* in the KO of ${V}_{K{O}_k}\cup i$ is lower (higher) than that in the KO of ${V}_{K{O}_k}$, we assign a positive (negative) sign to this edge.

In some cases, we may find more than one pair of KO experiments for an uncertain edge (*i,j*). For each of these KO pairs, we again employ a two-sample *t*-test with *α* = 0.01. The result of each *t*-test counts as a vote for the existence of the edge (*i,j*) in the case of rejection of the null hypothesis, or a vote against the existence of this edge in the case of failure to reject the null hypothesis. If the votes against the edge exceed those for the edge, then we remove this edge from the upper bound *G*^*U*^. Otherwise, we add the edge (*i,j*) to the lower bound *G*^*L*^. In case of a tie, we do not change the bounds, i.e. the edge (*i,j*) remains uncertain. We also determine the edge sign by voting. Specifically, we set a positive (negative) sign when the average expression of gene *j* in the KO of ${V}_{K{O}_k}\cup i$ is more frequently lower (higher) than that in the KO of ${V}_{K{O}_k}$, among the KO pairs giving confirmatory votes for the edge. When a tie occurs, we keep the original sign of the edge (*i,j*) from *G*^*U*^.

Once the ensemble bounds are updated, we recalculate the separatoids to reflect the changes brought by the additions and removals of edges to and from the bounds. We repeat the steps described above until we cannot find any suitable pairs of KO experiments for the remaining uncertain edges.

### Iterative gene regulatory network inference

**Fig. 3:**
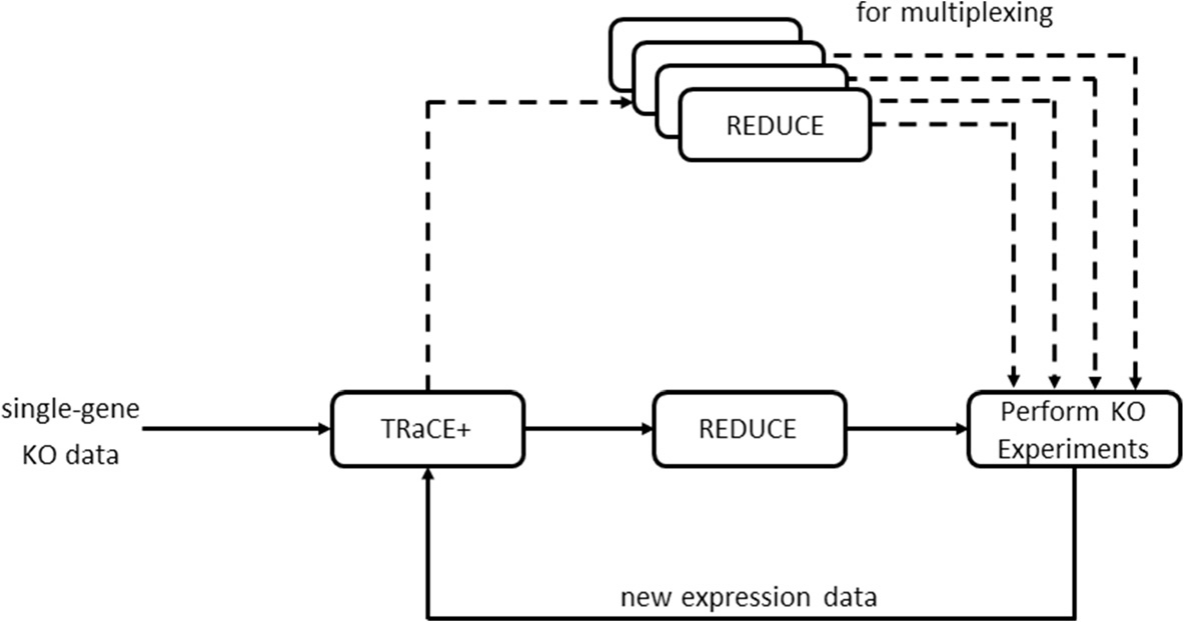
Iterative GRN inference procedure. The procedure was adapted from [15]. Dashed arrows indicate the procedure for using multiplexing assay

Recently, we proposed an iterative GRN inference procedure which combines TRaCE and an optimal design of gene KO experiments, called REDUCE (REDuction of UnCertain Edges) [15]. We demonstrated that this iterative procedure could resolve the underdetermined issue of the GRN inference, producing a unique GRN. TRaCE+ can substitute TRaCE in this iterative inference to enable the inference of a signed digraph model of GRN. As shown in Fig. 3, the iteration starts with the construction of signed digraphs of the ensemble bounds using single-gene KO data. Based on these bounds, we optimize the next set of gene KO experiments using REDUCE. Briefly, REDUCE employs the edge separatoids and a constrained optimization to obtain the optimal set of gene KOs that would enable the verification of the maximum number of uncertain edges. The next step in the iteration is to carry out the optimized gene KOs experiments and obtain new transcriptional expression data. Subsequently, we feed the data back to TRaCE+ to update the ensemble bounds following the procedure described in the previous section. We repeat these steps until the ensemble bounds converge or do not improve further, or until a given quota on the number of KO experiments has been reached.

If desired, one can also perform REDUCE multiple times in a given iteration to generate a large list of gene KO experiments (see Fig. 3, dashed arrows). This implementation is particularly suitable for using multiplexing assay technology. Here, at the end of one REDUCE optimization, we remove the set of verifiable uncertain edges from subsequent runs. We perform REDUCE until all uncertain edges become verifiable or until we hit constraint(s) on the optimization. When only a subset of these KO experiments could be performed, one should select gene KOs from the list above in the order that they are generated (since earlier runs of REDUCE are associated with more verifiable uncertain edges).

## Results

### Case studies and performance evaluation

In order to evaluate the performance of TRaCE+, we applied the algorithms to the ensemble inference of *Escherichia coli* GRN from single-gene KO experiments, and to the iterative inference of 100-gene gold-standard GRNs from DREAM 4 *in silico* network inference challenge [17, 18]. For each KO experiment, we generated 10 replicates of *in silico* (simulated) gene KO data using the benchmark GRN data generator GeneNetWeaver with the default parameters [17]. GeneNetWeaver uses a thermodynamic-based model of transcription and translation under independent and/or synergistic regulations [19]. The model consists of a system of stochastic differential equations (chemical Langevin equations), where the rates of change of concentrations for mRNA and protein are described by

$$ \begin{array}{c}\hfill {F}_i^{RNA}\left(x,y\right)=\frac{d{x}_i}{dt}={m}_i{f}_i(y)-{\lambda}_i^{RNA}{x}_i\hfill \\ {}\hfill {F}_i^{\Pr ot}\left(x,y\right)=\frac{d{y}_i}{dt}={r}_i{x}_i-{\lambda}_i^{\Pr ot}{y}_i\kern3.5em \hfill \end{array} $$

where, *x*_*i*_ and *y*_*i*_ are the mRNA and protein concentrations for gene *i*, respectively, *t* is time, *m*_*i*_ is the maximum transcription rate*, r*_*i*_ is the translation rate constant, *λ*_*i*_^*RNA*^ and *λ*_*i*_^Pr *ot*^ are the RNA and protein degradation rate constants, respectively. The function *f*_*i*_(*y*) describes the regulations of the transcription of gene *i* by different TF proteins. For example, the activation (positive regulation) of gene *k* expression by the protein product of TF gene *j* is described by a function *f*_*k*_(*y*), whose value increases with increasing value of *y*_*j*_. Further, in GeneNetWeaver, both the production and degradation of RNA and proteins are subjected to intrinsic stochastic noise, modeled as a random Wiener process [17]. Additionally, log-normal measurement noise is added to the simulated expression data [20]. In the case studies below, the KO of a gene *i* is simulated by setting the maximum transcription rate *m*_*i*_ to zero.

The quality of the ensemble bounds was assessed by using true positive rate, total distance and Jaccard distance with respect to the reference GRNs. The true positive rate (TPR) was calculated as the ratio between the number of edges in the reference GRN that were correctly identified in the lower bound *G*^*L*^ and the total number of edges in the reference network ${G}_{\varnothing}^{+}$, or more precisely:

$$ TPR=\frac{N\left(E\left({G}^L\right)\cap E\left({G}_{\varnothing}^{+}\right)\right)}{N\left(E\left({G}_{\varnothing}^{+}\right)\right)} $$

where *N*(*E*(${G}_{\varnothing}^{+}$)) denotes the cardinality of the set *E*(${G}_{\varnothing}^{+}$). Meanwhile, the total distance between the ensemble bounds and the reference GRN was computed as follows:

$$ TD=\frac{N\left(E\left({G}^U\right)\cup E\left({G}^L\right)\cup E\left({G}_{\varnothing}^{+}\right)\right)-N\left(E\left({G}^U\right)\cap E\left({G}^L\right)\cap E\left({G}_{\varnothing}^{+}\right)\right)}{N\left(E\left({G}_{\varnothing}^{+}\right)\right)} $$

A higher TD value indicates larger uncertainty in the GRN inference (i.e. worse inferability). Finally, the Jaccard distance (JD) was evaluated using the following formula:

$$ JD\left({G}_1,{G}_2\right)=\frac{N\left(E\left({G}_1\right)\cup E\left({G}_2\right)\right)-N\left(E\left({G}_1\right)\cap E\left({G}_2\right)\right)}{N\left(E\left({G}_1\right)\cup E\left({G}_2\right)\right)} $$

The JD gives a measure of similarity between two digraphs *G*_*1*_ and *G*_*2*_. A JD of 1 indicates that the two digraphs have no common edges and a JD of 0 implies that the two digraphs share the same set of edges. In the case studies, we evaluated the JDs between *G*^*U*^ and ${G}_{\varnothing}^{+}$, as well as between *G*^*L*^ and ${G}_{\varnothing}^{+}$. When dealing with ensemble bounds in the form of signed digraphs such as those generated by TRaCE+, the intersections among the sets of edges in the evaluations of TPR, TD and JD were done by respecting the sign of the edges (i.e. edges of unequal signs were not counted).

### Ensemble inference of *E. Coli* GRN

**Fig. 4:**
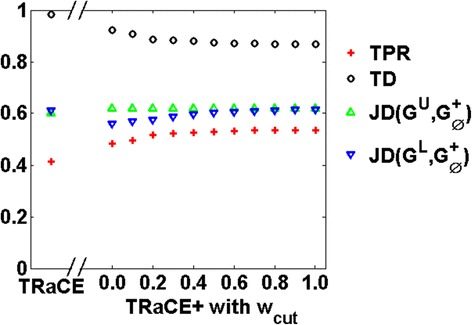
Ensemble bounds from TRaCE and TRaCE+ for *E. coli* GRN: True positive rate (TPR), total distance (TD) and Jaccard distance (JD)

In this case study, we used the signed digraph of *E. coli* GRN from GeneNetWeaver, containing 1565 genes and 3758 regulatory interactions [17]. We generated *in silico* data for the complete set of single-gene KOs as described in Methods. Using this dataset, we constructed unsigned digraph ensemble bounds using TRaCE [12] and signed digraph bounds using TRaCE+. For TRaCE+, we also studied how the ensemble bounds, particularly *G*^*L*^, depend on *w*_*cut*_ by varying this parameter between 0 and 1 (at 0.1 increments). We compared the performance of TRaCE and TRaCE+ according to the TPRs, TDs and JDs of the resulting bounds as described in Methods. The comparison in Fig. 4 shows that TRaCE+ could provide ensemble bounds with higher TPRs and lower JDs and TDs (of the *G*^*U*^ and *G*^*L*^ from ${G}_{\varnothing}^{+}$) than TRaCE. These trends demonstrated TRaCE+’s ability to extract information contained in the gene regulatory signs that were disregarded by TRaCE. The TDs of the bounds from TRaCE+ generally improved with higher values of *w*_*cut*_, but the improvements reached a plateau after *w*_*cut*_ of 0.4. However, the lower bounds from TRaCE+ slightly worsened with increasing *w*_*cut*_. The JDs between the upper bound and the reference network differed little between TRaCE+ and TRaCE due to the consideration of edge signs in computing JDs for the upper bounds from TRaCE+. As expected, the upper bound from TRaCE+ did not vary with *w*_*cut*_.

### Iterative GRN inference of DREAM4 100-gene networks

**Fig. 5:**
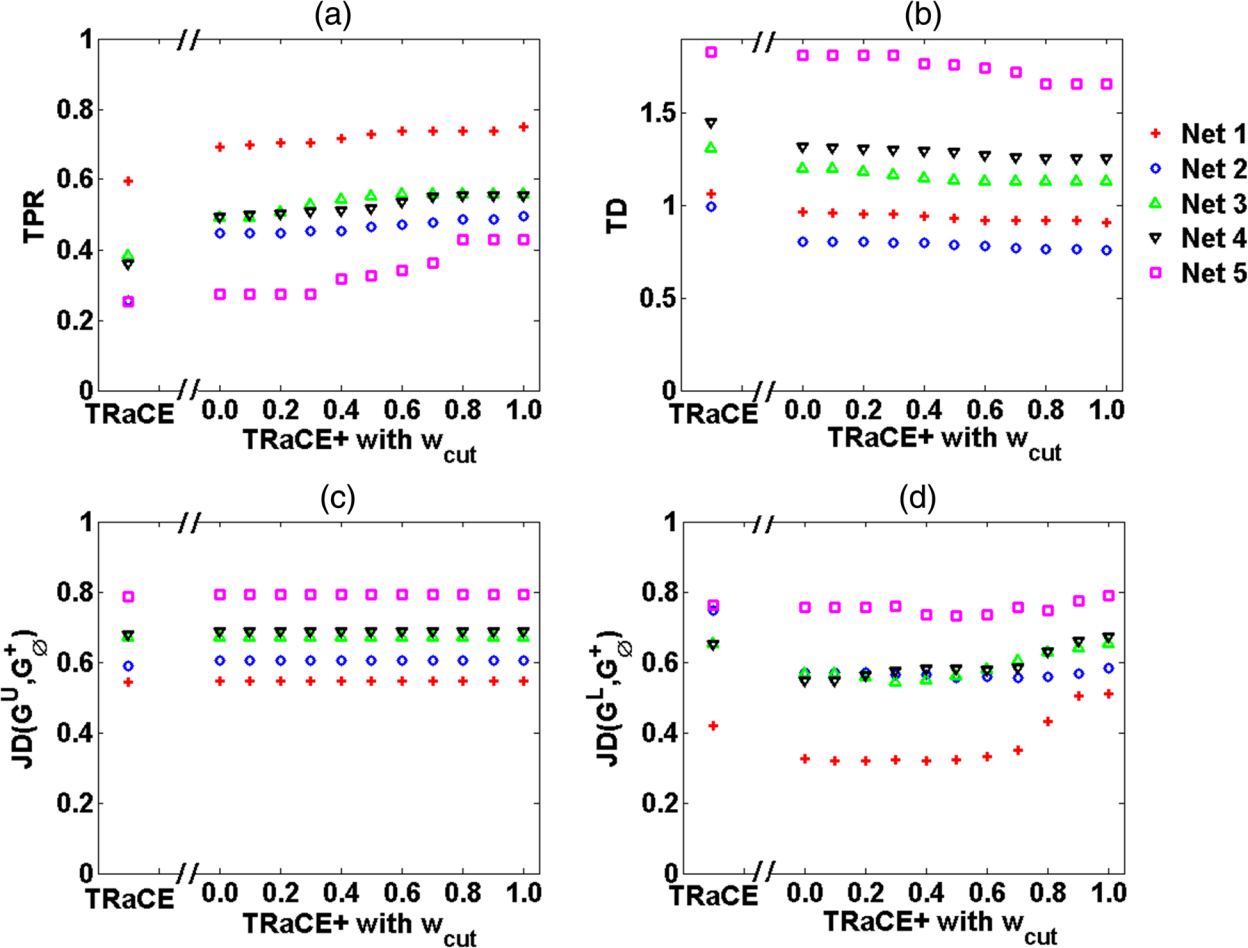
Ensemble bounds from TRaCE and TRaCE+ using single-gene KOs for DREAM 4 100-gene GRNs: **a** True positive rate (TPR), **b** total distance (TD), **c** Jaccard distance (JD) between *G* ^*U*^ and reference network, and **d** Jaccard distance (JD) between *G* ^*L*^ and reference network

In this case study, we applied the iterative GRN inference using either TRaCE or TRaCE+ to the five signed digraph gold-standard networks from DREAM 4 100-gene *in silico* network inference challenge [17, 18]. At the start of the iterative procedure, we simulated the complete set of single-gene KO data as described in Methods. Figure 5a and b show respectively the TPRs and TDs of the ensemble bounds. According to the TDs and TPRs, the ensemble bounds from TRaCE+ consistently outperformed those from TRaCE regardless of the parameter *w*_*cut*_. Here, TDs and TPRs improved slightly with increasing *w*_*cut*_. Figure 5c and d provide the JDs of the ensemble bounds from TRaCE and from TRaCE+ with different *w*_*cut*_ values. Like in the *E. coli* case study, the JDs of the ensemble upper bounds did not differ significantly between TRaCE and TRaCE+, nor did they depend on *w*_*cut*_. The JDs of the lower bounds from TRaCE+ were mostly better than those from TRaCE, where the best JDs corresponded to *w*_*cut*_ values between 0.2 and 0.4. In the following, we compared the performance of the iterative inference using TRaCE and using TRaCE+ with a *w*_*cut*_ of 0 (i.e. ignoring edge weights) and an intermediate *w*_*cut*_ of 0.3.

In the implementation of REDUCE, we put a constraint on the maximum number of genes in the optimal KO experiments. We started with a maximum of 2 genes, and incremented this constraint by 1 when the optimization within REDUCE could not produce any feasible solution. We again employed GeneNetWeaver to generate *in silico* data for the optimal KO experiments. We performed the iterative procedure until the ensemble bounds converged.

**Fig. 6:**
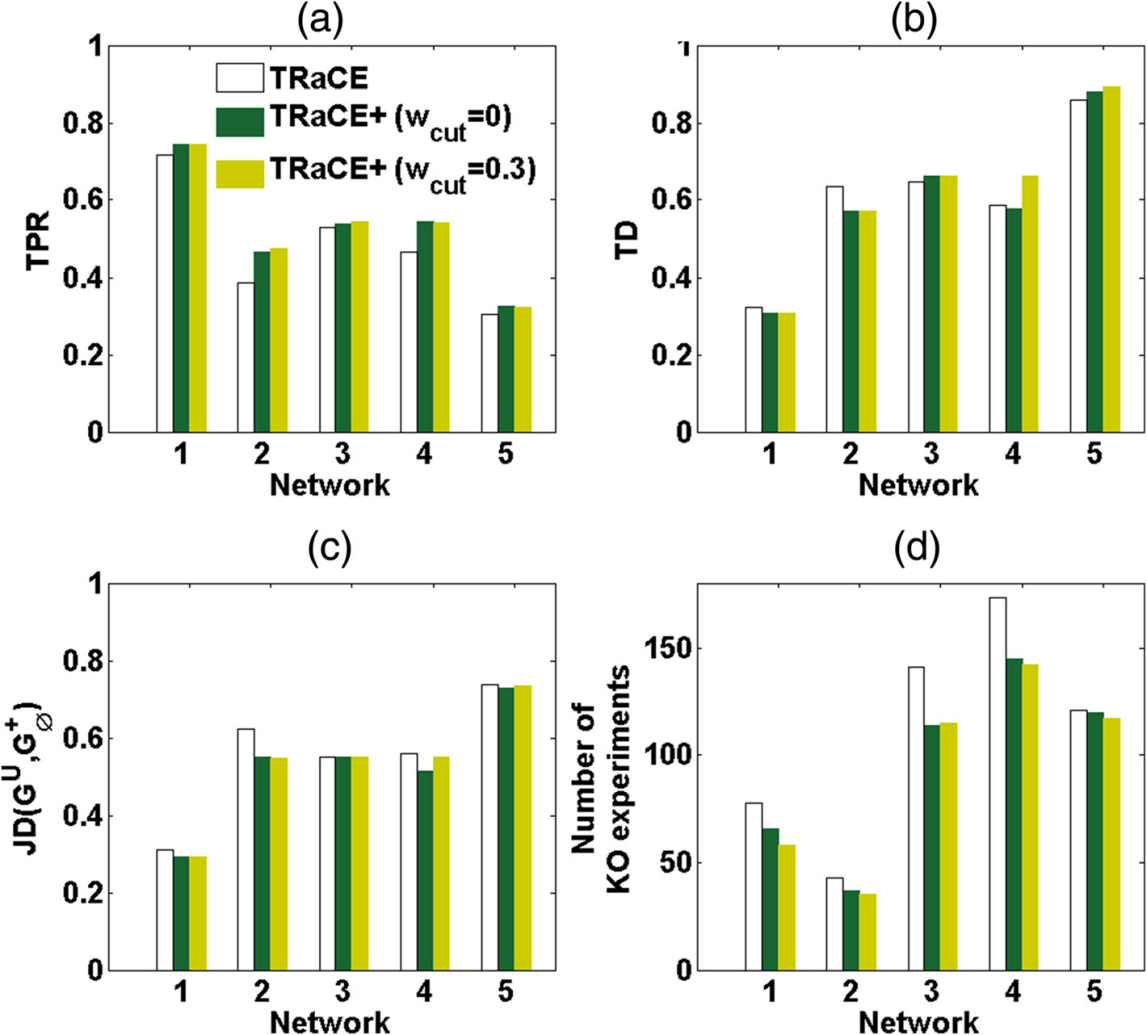
Comparison of iterative GRN inference using TRaCE and TRaCE+ (*w*_*cut*_ = 0 and *w*_*cut*_ = 0.3). **a** True positive rate (TPR), **b** total distance (TD), **c** Jaccard distance (JD), **d** total number of KO experiments

For all of the five gold-standard GRNs, the iterations terminated in the convergence of the ensemble bounds, i.e. we obtained a unique GRN. Figure 6 shows the TPRs, TDs and JDs of the inferred GRNs, as well as the total number of KO experiments required (excluding single-gene KOs). The iterations using TRaCE+ (*w*_*cut*_ = 0 and *w*_*cut*_ = 0.3) produced slightly better GRNs than TRaCE in terms of TPRs and JDs. Of course, the edges in the GRNs from TRaCE+ had signs, while those from TRaCE did not. More importantly, the iterations using TRaCE+ required much fewer KO experiments to reach convergence than TRaCE (*p* = 0.013 *w*_*cut*_ = 0 and *p* = 0.027 for *w*_*cut*_ = 0.3), by as much as 19 %. This trend signified the ability of TRaCE+ to extract more information from the data. Table 1 further compares the number of iterations and the highest number of genes involved the KO experiments. The numbers of iterations using TRaCE+ were generally lower than using TRaCE.

**Table 1:** Iterative inference of DREAM4 100-gene gold-standard GRN using TRaCE and TRaCE+ (*w*_*cut*_ = 0 and *w*_*cut*_ = 0.3): Number of iterations and maximum number of genes in KO experiments

Gold-standard Network	TRaCE		TRaCE+ (*w*_*cut*_ = 0)		TRaCE+ (*w*_*cut*_ = 0.3)	
	Iterations	Max KO	Iterations	Max KO	Iterations	Max KO
1	34	3	31	3	27	4
2	21	2	18	2	17	2
3	55	3	49	3	51	3
4	80	3	70	3	68	4
5	54	3	56	3	54	3

**Fig. 7:**
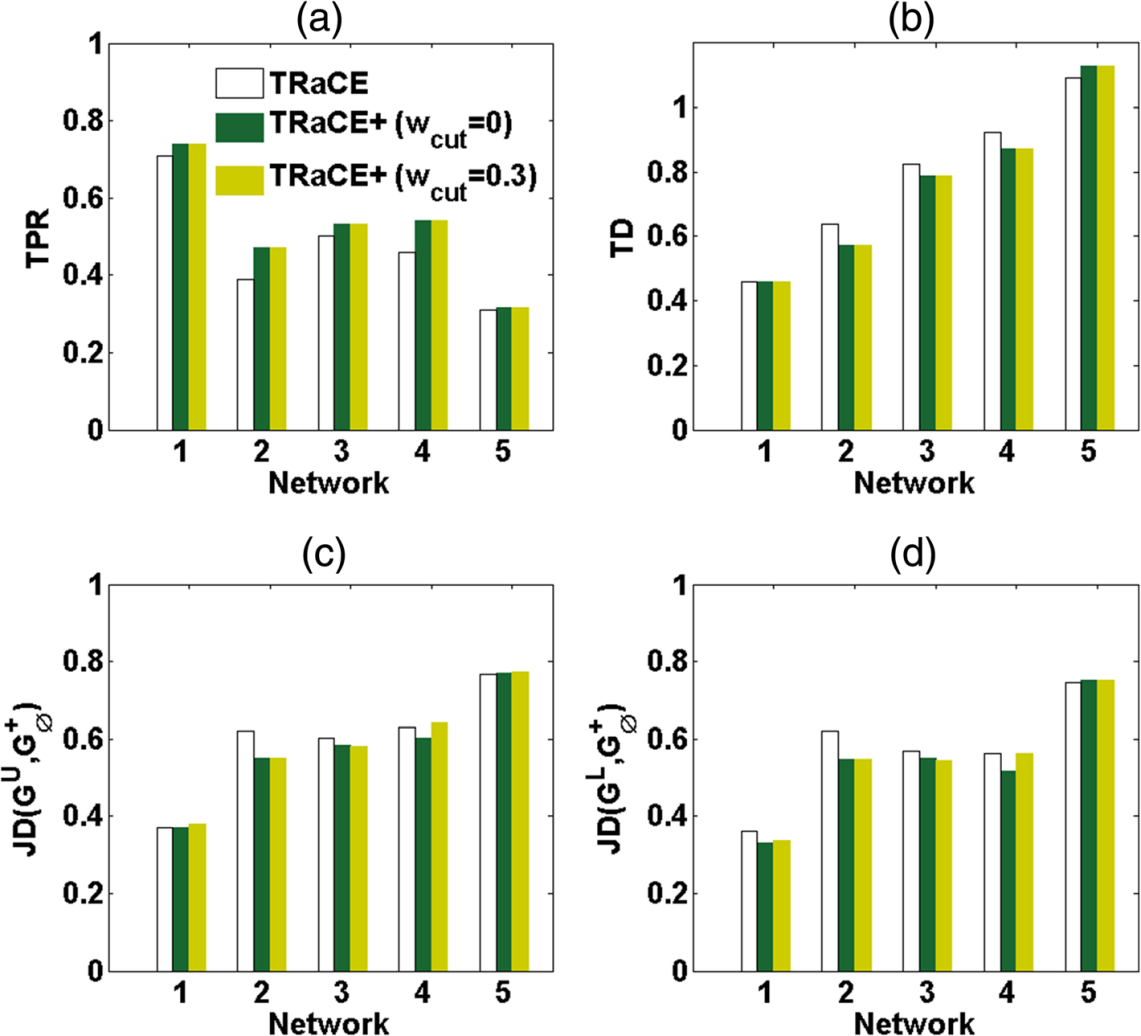
Comparison of ensemble bounds updates using the complete double-gene KO data in TRaCE and TRaCE+ (*w*_*cut*_ = 0 and *w*_*cut*_ = 0.3). **a** True Positive Rate (TPR), **b** Total Distance (TD), **c** Jaccard distance (JD) between *G* ^*U*^ and reference network, and **d** Jaccard distance (JD) between *G* ^*L*^ and reference network

As a further comparison, we also generated *in silico* data for the complete set of double-gene KOs, a total of 4,950 KO experiments. We used this dataset to update the ensemble bounds initially constructed using single-gene KOs. As shown in Table 2, only a small fraction of the double-gene KO experiments were useful for verifying uncertain edges, and a number of uncertain edges still remained after the ensemble bound update. Figure 7 gives the TPRs, TDs and JDs of the ensemble bounds. Compared to TRaCE, the bounds update considering edge signs in TRaCE+ led to better TPRs (*p* = 0.02 for *w*_*cut*_ = 0, and *p* = 0.016 for *w*_*cut*_ = 0.3). However, the differences in the TDs and JDs between TRaCE and TRaCE+ were not significant.

**Table 2:** Ensemble bound update using double-gene KOs in TRaCE and TRaCE+ (*w*_*cut*_ = 0 and *w*_*cut*_ = 0.3): Number of informative double-gene KOs for verifying uncertain edges and number of remaining uncertain edges after ensemble bound update

Network	TRaCE		TRaCE+ (*w*_*cut*_ = 0)		TRaCE+ (*w*_*cut*_ = 0.3)	
	Informative experiments	Uncertain edges	Informative experiments	Uncertain edges	Informative experiments	Uncertain edges
1	36	15	24	20	20	21
2	27	1	24	1	22	1
3	61	37	52	30	50	27
4	49	81	43	67	27	92
5	46	37	44	37	44	37

**Fig. 8:**
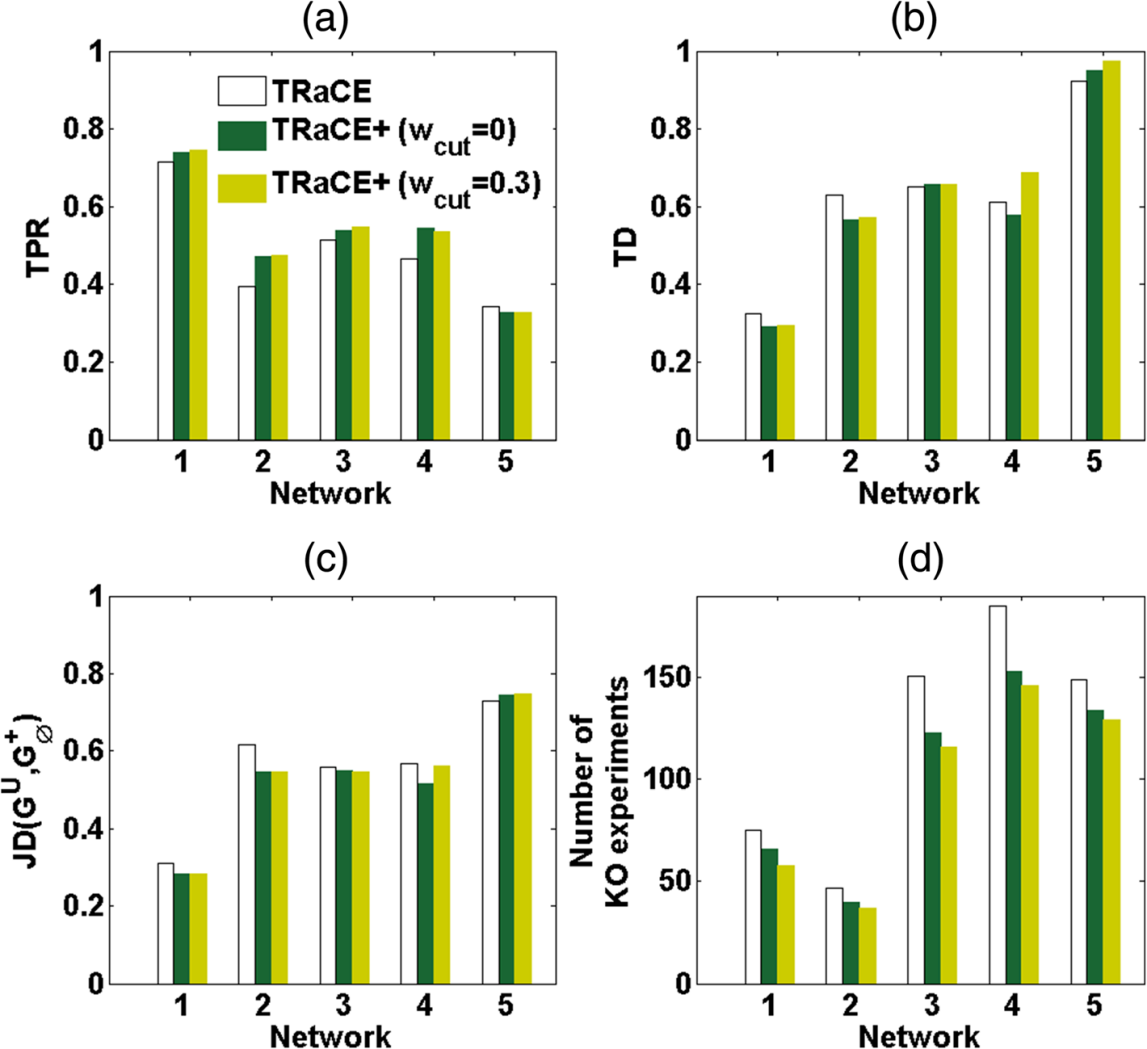
Comparison of iterative GRN inference with multiplexing assay using TRaCE and TRaCE+ (*w*_*cut*_ = 0 and *w*_*cut*_ = 0.3). **a** True positive rate (TPR), **b** total distance (TD), **c** Jaccard distance (JD), and **d** total number of KO experiments

Finally, we performed the iterative procedure using TRaCE and using TRaCE+ with *w*_*cut*_ = 0 and *w*_*cut*_ = 0.3, where we implemented multiplexed REDUCE to generate a large number of KO experiments. We again fixed the maximum number of genes in the KO experiments at each iteration, beginning with 2 and incrementing this limit by 1 when multiplexed REDUCE could not generate any feasible KO experiments. For all gold-standard networks, the iterations generated a unique GRN (i.e. the ensemble bounds converged). Fig. 8 summarizes the quality of the ensemble bounds according to TPRs, TDs and JDs. The results closely resembled those from the iterations without multiplexing. Again, employing TRaCE+ led to fewer total KO experiments than using TRaCE (*p* = 0.011 *w*_*cut*_ = 0 and *p* = 0.006 for *w*_*cut*_ = 0.3). In comparison to the results without multiplexing in Table 1, Table 3 shows that multiplexing could reduce the number of iterations tremendously.

**Table 3:** Iterative inference of DREAM4 100-gene gold-standard GRNs with multiplexing assay using TRaCE and using TRaCE+ (*w*_*cut*_ = 0 and *w*_*cut*_ = 0.3): Number of iterations and maximum number of genes in KO experiments

Net	TRaCE		TRaCE+		TRaCE+(w)	
	Iterations	Max KO	Iterations	Max KO	Iterations	Max KO
1	7	3	5	3	6	4
2	5	2	4	2	4	2
3	10	3	11	3	12	3
4	14	3	15	3	14	3
5	11	3	12	4	13	4

## Discussion

In this work, we developed a new ensemble inference algorithm called TRaCE+ for the identification of GRN structures in the form of a signed digraph. Unlike the traditional GRN inference, TRaCE+ produces upper and lower bounds of an ensemble of signed digraphs, describing the family of GRNs that are consistent with the gene accessibility relationships established by the input transcriptional expression profiles. Specifically, these bounds define the set of uncertain gene regulatory edges that could not be verified by the available data. The outputs of TRaCE+ are directly compatible with our recent optimal design of gene KO experiments called REDUCE and the accompanying iterative GRN procedure [15]. As shown in the case study using 100-gene gold-standard GRNs from DREAM 4 *in silico* network inference challenge, by iterating TRaCE+, REDUCE and performing optimized gene KO experiments, one can overcome the underdetermined issue of GRN inference and obtain a unique GRN in a relatively small number of iterations (especially when using multiplexing assay). Like TRaCE, a drawback of TRaCE+ is that the procedure requires at the minimum the complete set of single-gene KO data, which could become prohibitive for large-scale GRNs. If the TFs are known, then the requirement reduces to single-gene KOs of the TF genes. Nevertheless, we expect that accelerating progress in high-throughput gene editing technology (e.g., CRISPR-Cas9) and RNA sequencing will soon make this requirement non-limiting.

The consideration of regulatory signs in TRaCE+ represents a significant advance over TRaCE, as the mode of the gene regulations (activation/repression) is very often an important aspect in the applications of GRN. For example, when the inferred GRN is used in finding treatment of diseases or in drug discovery, the precise knowledge on the modes of the gene regulations matters tremendously. At the same time, the computational challenge arising from accounting the signs of the regulatory edges in the GRN digraph was not trivial. The issue of sign consistency could severely complicate performing transitive reduction [13], a key step in TRaCE+. Here, we adapted LTR to get around the issue in obtaining transitive reductions for GRNs with negative cycles. As demonstrated in the case studies, by taking the edge signs into account, TRaCE+ can extract more information from the data than TRaCE. As a result, the numbers of uncertain edges in the ensemble from TRaCE+ were consistently lower than those from TRaCE using the same set of KO data (single-gene KOs). Furthermore, in the iterative inference, employing TRaCE+ led to significantly fewer total gene KO experiments to reach convergence than using TRaCE. While we used edge weights only for constructing the ensemble lower bound from the initial upper bound, these weights could also serve as a measure of confidence (likelihood) for the existence of an edge (as done in a previous method called TRANSWESD [13]).

There exist many reasons for errors to happen during the ensemble bounds construction and updates, including noise and bias in expression data as well as (type-I and type-II) errors in the statistical tests. Three types of errors can appear in the ensemble bounds from TRaCE+. False negative (FN) errors involve regulatory edges in the reference GRN that do not appear in the upper bound *G*^*U*^. Meanwhile, false positive (FP) errors pertain to regulatory edges in the lower bound *G*^*L*^ that do not belong to the true network. Finally, incorrect sign (IS) errors refer to edges in the reference GRN that have the opposite signs in the upper bound. Among the three types of errors, our experience from the case studies showed that FNs were the most common errors while IS errors were the least common, typically affecting less than 1 % of the edges in the reference networks. We further noted that the majority (>80 %) of FN errors in the case studies were associated with fan-in motifs, where several genes regulated a common target gene. Here, knocking-out only one of the regulators might not cause any differential expression of the target gene due to compensation by the other regulator(s).

Once occurred, FP and FN errors could not be corrected during the iterative GRN inference since these errors affected edges that were *not* uncertain. In the second case study, the large majority of the errors in the inferred GRN were already present in the initial ensemble bounds constructed using single-gene KO data. Nevertheless, new FP and FN errors could also appear and accumulate over the iterations. By modifying the parameters in TRaCE+, including *z*_*cutoff*_, *z*_*threshold*_, *w*_*cut*_ and *α*, we can adjust the frequency of FPs and FNs. Lowering *z*_*cutoff*_ and *z*_*threshold*_ has the effect of reducing FN errors, but comes at the cost of higher FP errors and uncertain edges. We previously showed that *z*_*cutoff*_ = 3 and *z*_*threshold*_ = 2 provide a good balance among FNs, FPs and uncertain edges [12]. Meanwhile, increasing *w*_*cut*_ could reduce the number of uncertain edges, but also cause more FPs. On the other hand, lowering the parameter *α* in the ensemble bound update should reduce FPs at the trade-off of increasing FNs. In the ensemble bounds of *E. coli* and DREAM 4 GRNs from single-gene KO data, the frequency of FNs ranged between 24 and 56 % (*E. coli:* 44 %), while the frequency of FPs varied between 2.8 and 13 % (*E. coli*: 8.1 %) when using *w*_*cut*_ = 0. These frequencies were reported as a fraction of the number of edges in the reference GRN. Increasing *w*_*cut*_ to 0.3 led to more FPs, especially for *E. coli* GRN (from 8.1 to 26 %). The increase in FPs by using *w*_*cut*_ = 0.3 among DREAM 4 GRNs was however more modest (2.9 %–14.5 %).

In this study, we focused specifically on transcriptional expression data from gene KO experiments when creating the ensemble of GRNs. Nevertheless, other types of information, such as transcription factor binding sites (TFBS) from chromatin immunoprecipitation-sequencing (ChIP-seq), ChIP-chip, and/or cap analysis gene expression (CAGE) data, could also be used to refine the ensemble bounds. For example, we could verify uncertain edges emanating from a TF based on the existence or absence of its binding site in the promoter region of a target gene. In addition, TFBS could also be used to identify and correct FPs and FNs. However, the identification of the target genes of a TF based on ChIP-seq, ChIP-chip and CAGE data is not error-free. Thus, some care has to be taken to avoid accumulating different sources of errors. An integrative analysis of different types of data for ensemble GRN inference is out of the scope of this work, but is a topic of particular interest in our groups.

## Conclusion

The inference of an ensemble of networks, rather than a single network, provides an avenue to cope with the underdetermined nature of the GRN inference from transcriptional expression data. In this work, we developed TRaCE+ for the generation of upper and lower bound signed digraphs of GRN ensemble from gene KO data. TRaCE+ significantly expanded the capability of our previous method TRaCE, enabling the inference of the mode of the gene regulations by considering the signs of the regulatory edges. As demonstrated in the case studies, TRaCE+ could extract more information from gene KO data than TRaCE, and as a result, reduce the number of uncertain edges. When employed within an iterative inference procedure, TRaCE+ required much fewer KO experiments to identify a unique GRN than TRaCE, and slightly improved the quality of the reconstructed networks.

## Abbreviations

CAGE, cap analysis gene expression; ChIP, chromatin immunoprecipitation; ChIP-seq, chromatin immunoprecipitation - sequencing; ConTREx, condensation, transitive reduction, and expansion; DAG, directed acyclic graph; DNA, deoxyribonucleic acid; DREAM, dialogue on reverse-engineering assessment and methods; FN, false negative; FP, false positive; GRN, gene regulatory network; IS, incorrect sign; JD, jaccard distance; KO, knock-out; LTR, local transitive reduction; REDUCE, reduction of uncertain edges; RNA, ribonucleic acid; TD, total distance; TF, transcription factor; TFBS, transcription factor binding sites; TPR, true positive rate; TRaCE, transitive reduction and closure ensemble; TRANSWESD, transitive reduction for weighted signed digraphs
